# Preliminary Results on the Use of Leather Chrome Shavings for Air Passive Sampling

**DOI:** 10.1155/2012/897872

**Published:** 2012-07-24

**Authors:** D. Sanjuán-Herráez, L. Chabaane, S. Tahiri, A. Pastor, M. de la Guardia

**Affiliations:** ^1^Department of Analytical Chemistry, University of Valencia, Research Building, 50th Dr. Moliner Street, 46100 Burjassot, Spain; ^2^Département de Chimie, Faculté des Sciences d'El Jadida, Université Chouaïb Doukkali, B.P. 20, 24000 El Jadida, Morocco

## Abstract

A new passive sampler based on low-density polyethylene (LDPE) layflat tube filled with chrome shavings from tannery waste residues was evaluated to determine volatile organic compounds (VOCs) in indoor and outdoor areas. VOCs were directly determined by head space-gas chromatography-mass spectrometry (HS-GC-MS) without any pretreatment of the sampler and avoiding the use of solvents. Limit of detection values ranging from 20 to 75 ng sampler^−1^ and good repeatability values were obtained for VOCs under study with relative standard deviation values from 2.8 to 9.6% except for carbon disulfide for which it was 22.5%. The effect of the amount of chrome shavings per sampler was studied and results were compared with those obtained using empty LDPE tubes, to demonstrate the capacity of chrome shavings to adsorb VOCs.

## 1. Introduction

The operations involved in the transformation of hides into leather generate both liquid and solid pollution loads at various processing stages [1]. The processing of one metric ton of raw hide provides 200 kg of a leather-final product, along with 250 kg of nontanned waste, 200 kg of tanned waste, and 50.000 kg of wastewater. Thus, only 20% of the raw material weight is converted to leather [2]. The World Bank reported that solid wastes can represent up to 70% of the wet weight of the original hides [3]. Tanned solid wastes (as chrome shavings) are of low density and therefore occupy a large volume. It causes problems in the handling of enormous wastes generated in leather industry. Wastes of chromium-tanned leather primarily consist of chromium and proteins. These wastes are stables and not subject to putrefaction. The biological stability of the material is the result of complexation between chromium (III) salts and the carboxyl groups of the collagen. According to the literature, there are several studies focused on the valorization of the aforementioned solid wastes as the production of activated carbon from chromium- and vegetable-tanned leather shaving wastes [4], treatment and cleaning of water polluted by oils [5], hydrocarbons [6], and organic dyes [7] using chrome shavings, the use of chestnut and mimosa tannins immobilized on chrome shavings matrices as adsorbents for the recovery of Cr (VI) from polluted aqueous systems [8], the use of wet blue chrome shavings as thermal insulator [9], and so forth.

Semipermeable membrane devices (SPMDs) were introduced by Huckins et al. [10], initially, as passive water samplers [11], and, subsequently, Petty et al. [12] proposed the use of SPMDs for the air sampling of pollutants. A SPMD consists of a LDPE layflat tube filled with triolein and sealed at both ends. Recently, a versatile, easy and rapid atmospheric monitor (VERAM) new type of passive samplers was developed by our group [13], where a solid phase was employed instead of triolein as filler of LDPE layflat tubes. VOCs [14] and pesticides [15] were successfully determined by the use of VERAM samplers filled with activated carbon and florisil in air of indoor areas.

In this study, a new passive sampler based on the use of solid tannery wastes (chrome shavings) as filler has been evaluated as a green and cheap alternative to other samplers in order to evaluate the presence of 16 VOCs in air. VOCs retained in the samplers were directly determined without any sample pretreatment and avoiding the use of solvents by head space-gas chromatography-mass spectrometry (HS-GC-MS) in only 20 min.

## 2. Material and Methods

### 2.1. Apparatus and Reagents

Head space-gas chromatography-mass spectrometry (HS-GC-MS) direct analysis of devices was performed using an HS2000 injector from Finnigan (Waltham, MS, USA), a Finnigan Trace gas chromatograph equipped with low-bleed HP (30 m × 0.32 mm, 0.25 *μ*m) capillary column, and Finnigan Polaris Q ion trap mass spectrometer detector.

LDPE layflat tubing with 2.9 cm wide, obtained from Garciplast (Barcelona, Spain) and chrome shavings obtained from a tannery plant (Mohammedia, Morocco) were employed to prepare the passive samplers. (It must be noticed that shavings of wet blue leather have a highly organized structure in the form of fibers (*ϕ* = 100 nm) which are parallel and very tight to each other and that they have an important percentage of proteins (78.64%) and a chromium percentage of about 3% [7]). A Roblevoc sealer (Barcelona, Spain) was used to heat-seal the membranes after to be filled with the solid phase.

Studied VOCs include carbon disulfide; chloroform; 1,1,1-trichloroethane; 1,2-dichloroethane; benzene; cyclohexane; 2,2,4-trimethylpentane; trichloroethylene; bromodichloromethane; toluene; dibromochloromethane; tetrachloroethylene; ethylbenzene; *m*, *p*-xylene; o-xylene and bromoform standards and *n*-hexadecane were obtained from Scharlau (Barcelona, Spain), Fluka Chemie (Buchs, Switzerland), Merck (Darmstadt, Germany), and Sigma-Aldrich (St. Louis, USA). Toluene-d8 (99.96%) from Aldrich (Steinheim, Germany) was used as internal standard (IS) at a final concentration of 300 ng in *n*-hexadecane.

VOCs accumulated in passive samplers were directly determined using glass vials, with an internal volume of 10 mL, capped with PTFE-butyl rubber seals for HS measurements.

### 2.2. Preparation of Passive Samplers

**Figure 1:**
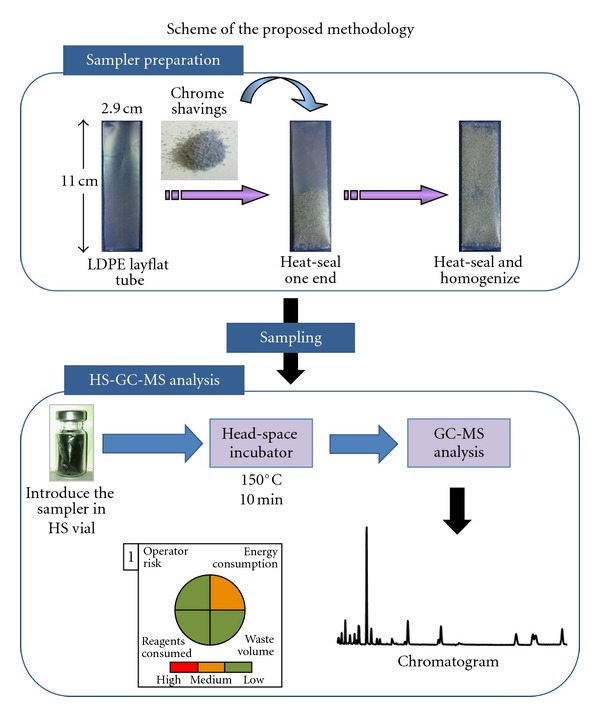
Chrome shavings based passive sampler preparation and HS-GC-MS analytical method employed for the determination of VOCs. Inset 1 : pictogram describing the green parameters of the employed method.

LDPE layflat tubing was cut in 10 cm segments which were soaked overnight in hexane in order to remove any possible additives and interfering compounds, according to the previously reported methods [14, 16]. Then, one end was heat-sealed and an amount of chrome shavings was introduced into the LDPE, shaking the device to extend the solid phase over the entire surface, and finally, the other end was also heat-sealed, with a final effective length of 9 cm (see Figure 1). Previously, chrome shavings were placed in the oven at 105°C during 5 hours, in order to evaporate the possible water content. All samplers were separately wrapped in aluminium foils and stored in closed vessels at −20°C until their use to avoid contaminations.

Passive samplers were filled with different amounts of chrome shavings in order to found the best analytical response. Studied amounts were 25, 50, 100, and 150 mg.

### 2.3. Deployment of Passive Samplers

Passive samplers filled with different amounts of chrome shavings and LDPE membranes without any filler were deployed during 24 h in different glass containers to sample air spiked with 5.26 mg m^−3^ VOCs.

### 2.4. HS-GC-MS Determination

Deployed and spiked passive samplers were heated at 150°C for 10 min in the HS oven, and a volume of 0.1 mL vapour phase generated inside glass vials was injected in the GC-MS system, with a syringe temperature of 150°C and a constant air flow purge. Injection was done in split mode (1 : 10) at 200°C, employing 1.3 mL min^−1^ constant flow of helium as carrier gas. The GC oven temperature program was 40°C, held for 9 min, increased at a rate of 20°C min^−1^ up to 200°C, and finally held for 2 min. The transfer line and source temperatures were 280 and 250°C, respectively. Electron impact ionization at 70 eV was employed and a mass scanning range from 40 to 200 *m*/*z* was used for full scan acquisitions. The measured *m*/*z* ions employed for each compound are shown in Table 1, together with their chromatographic retention times, being selected deuterated toluene as internal standard (IS), due to its intermediate position between the studied compounds.

## 3. Results and Discussion

### 3.1. Evaluation of Passive Samplers

**Figure 2:**
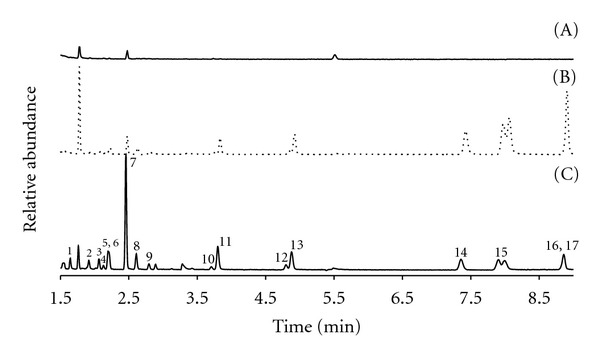
Total ion chromatogram obtained for a blank (A), a passive sampler deployed 24 h in a glass container with air spiked at 5.26 mg m^−3^ VOCs level (B), and a passive sampler spiked with 2.5 *μ*g of VOCs standard solution (C). Note: peaks correspond to carbon disulfide (1); chloroform (2); 1,1,1-thrichloroethane (3); 1,2-dichloroethane (4); benzene (5); cyclohexane (6); 2, 2, 4-thrimethylpentane (7); trichloroethylene (8); bromodichloromethane (9); toluene-d8 (10); toluene (11); dibromochloromethane (12); tetrachloroethylene (13); ethylbenzene (14); *m*, *p*-xylene (15); *o-*xylene (16); bromoform (17).

Figure 2 shows the total ion chromatograms obtained for deployed and spiked passive samplers filled with 150 mg of chrome shavings, and it can be seen that the tannery wastes present a high capability to catch polar and nonpolar compounds. However, for quantitative measurements, extracted ion chromatograms were used for each VOC compound based on the selection of ions indicated in Table 1.

**Figure 3:**
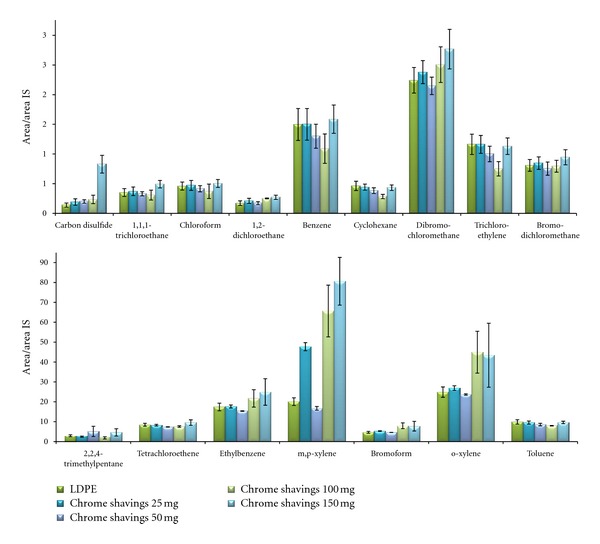
HS-GC-MS relative signals with standard deviations obtained from chrome shaving passive samplers deployed on air spiked with 5.26 mg m^−3^ VOCs as a function of the use of different amounts of chrome shavings and empty LDPE. The deployment time was 24 h in all cases.

On the other hand, studies based on the use of deployed passive samplers containing different amounts of chrome shavings (see Figure 3) suggest that the use of chrome shavings improves the signal response obtained with empty LDPE, although differences are not too big for all compounds. Carbon disulfide, chloroform, dibromochloromethane, ethylbenzene, 2,2,4-trimethylpentane, and xylenes show better results than empty membranes with a clear improvement on the analytical signals, whereas benzene, bromoform, 1,2-dichloroethane, and 1,1,1-trichloroethane shows a slight improvement on the HS-GC-MS signals. The remaining VOCs show similar behaviour that those obtained in the absence of chrome shavings.

From data reported in Figure 3, it can be concluded that 150 mg chrome shavings provides the best results for VOCs retention. Thereby, analytical features were established for passive samplers filled with 150 mg of chrome shavings. Higher amounts of filler material were not studied because of the difficulty to roll up and introduce the deployed passive samplers inside 10 mL HS glass vials. Blanks of both, LDPE and passive sampler filled with leather chrome shavings were measured in order to ensure that LDPE and chrome shavings were not contaminated.

### 3.2. Analytical Features

The HS relative signal of each VOC under study was calculated by comparing the chromatographic peak areas from an injection of a passive sampler spiked with 2.5 *μ*g VOCs with those obtained for the same amount of VOCs measured in the absence of sampler. Results obtained ranged from 34.8 to 65.1% (see Table 1) which indicates that chrome shavings provide a strong retention of VOCs. So, their use in passive samplers reduces the sensitivity to be obtained and requires to prepare the calibration with HS vials loaded with the samplers.

Calibration curves were established with passive samplers spiked with VOC standards dissolved in *n*-hexadecane, at five concentration levels, from 20 ng to 10 *μ*g. Limit of detection (LOD) values were established as 3 times the signal to noise ratio provided by the signals found for the minimum amount of VOC present in the sampler. This ratio was calculated by Xcalibur software provided by Thermo-Finnigan equipment. Limit of quantification (LOQ) values were calculated as 10 times the standard deviation of the measurements of the lowest standard.

Table 2 shows correlation coefficients (*r*), LODs, LOQs, reproducibility and relative standard deviation (RSD) values obtained for each compound under study. Correlation coefficients varied from 0.993 to 0.99998. These values suggest a good linearity along the studied concentration range for all studied compounds. LOD values achieved ranged from 20 to 75 ng VOCs per sampler and LOQ values ranged from 60 to 150 ng VOCs per sampler, which is good enough to detect the presence of these compounds in polluted areas. Precision expressed as reproducibility was calculated as $S/\bar{x}$, where *S* is the standard deviation of three consecutive injections of three different samplers spiked with 2.5 *μ*g VOC standard mixture and $\overset{-}{x}$ is the mean of the aforementioned injections. Values obtained varied from 2.8 to 9.6% except for carbon disulfide for which this parameter was 22.5%.

RSD values were obtained as $S/\overset{-}{x}$, where *S* is the standard deviation of three consecutive injections of the same sampler spiked with 2.5 *μ*g VOC standard mixture and $\overset{-}{x}$ is the mean of the aforementioned injections with a cooling time of 30 min between each injection, in order to evaluate the possibility to do replicate analysis of the same sampler. Results obtained show values from 1.7 to 7.9% except for carbon disulfide for which RSD was 20%. These results confirm the possibility to do several injections of the same sampler for every studied VOC, thus contributing to obtain as much as possible information from the samplers.

## 4. Conclusions

A new passive sampler, based on the use of LDPE layflat tubes filled with leather chrome shavings as solid phase, was evaluated to determine VOCs in air. Results obtained show that the use of chrome shavings improves the signal response obtained for empty LDPE membranes. Due to the high quantities of solid wastes generated by the tanning industries, this is a cheap and green alternative to other solid phases as triolein, tenax, activated carbon, florisil, and so forth, to build passive samplers. Moreover, the simplicity of the analytical methodology proposed (HS-GC-MS) and the fact that this procedure does not require any sample pretreatment, also avoiding the use of solvents, make this procedure a green analytical alternative for the determination of VOCs in air [17].

## Figures and Tables

**Table 1:** GC-MS measurement parameters of VOCs under study.

Compound	Rt^a^ (min)	Measurement ions (m/z)	HS relative signal (%)
Carbon disulfide	1.55	76	34.8
Chloroform	1.91	83 + 85	42.9
1,1,1-Trichloroethane	2.07	97 + 99	42.3
1,2-Dichloroethane	2.13	62 + 64	41.8
Benzene	2.19	78 + 77	44.5
Cyclohexane	2.22	56 + 84	45.8
2,2,4-Trimethylpentane	2.46	41 + 57	56.3
Trichloroethylene	2.61	132 + 130	47.0
Bromodichloromethane	2.79	85 + 83	44.8
Toluene-d8^b^	3.71	98 + 100	—
Toluene	3.80	91 + 92	58.7
Dibromochloromethane	4.80	127 + 129	51.3
Tetrachloroethylene	4.88	166 + 164	65.1
Ethylbenzene	7.36	91 + 106	64.4
*m*,*p*-Xylene	7.95	91 + 106	64.3
*o*-Xylene	8.85	91 + 106	48.5
Bromoform	8.86	173 + 175	64.0

^a^Retention time.

^b^Internal standard.

**Table 2:** Analytical features of the HS-GC-MS determination of studied VOCs using chrome-shavings-based passive samplers.

Compound	*r* ^ a^	LOD^b^ (ng sampler^−1^)	LOQ^c^ (ng sampler^−1^)	Reproducibility^d^ (%)	RSD^e^ (%)
Carbon disulfide	0.99904	20	60	22.5	20.0
Chloroform	0.99323	75	120	9.6	2.1
1,1,1-Trichloroethane	0.99995	20	80	8.9	5.4
1,2-Dichloroethane	0.99998	75	110	9.3	7.9
Benzene	0.99973	75	100	5.1	3.4
Cyclohexane	0.99852	75	120	4.3	4.6
2,2,4-Trimethylpentane	0.99973	20	60	6.2	4.3
Trichloroethylene	0.99930	75	150	2.9	2.6
Bromodichloromethane	0.99620	75	130	4.9	2.2
Toluene	0.99903	20	60	2.8	1.7
Dibromochloromethane	0.99868	75	150	3.9	1.8
Tetrachloroethene	0.99967	20	70	4.6	2.8
Ethylbenzene	0.99835	20	70	7.0	6.3
*m,p*-Xylene	0.99842	20	80	8.0	6.8
Bromoform	0.99858	75	150	7.0	6.0
*o*-Xylene	0.9994	20	80	9.5	7.0

^a^Correlation coefficients.

^b^Limit of detection.

^c^Limit of quantification.

^d^Reproducibility at 2.5 *μ*g spiked level (*n* = 3).

^e^Relative standard deviation at 2.5 *μ*g spiked level (*n* = 3).
